# Predicting sight-threatening diabetic retinopathy using the combination of serum cystatin C and indirect bilirubin

**DOI:** 10.3389/fendo.2026.1751821

**Published:** 2026-03-19

**Authors:** Yujie Yan, Lulu Song, Yangyang Xu, Shancheng Si

**Affiliations:** 1 Department of Ophthalmology, China-Japan Friendship Hospital, Beijing, China; 2 Department of Endocrinology, China-Japan Friendship Hospital, Beijing, China; 3 Beijing Tsinghua Changgung Hospital Eye Center, Beijing Visual Science and Translational Eye Research Institute (BERI), Tsinghua Medicine, Tsinghua University, Beijing, China

**Keywords:** biomarkers, cystatin C, indirect bilirubin, sight-threatening diabetic retinopathy, type 2 diabetes mellitus

## Abstract

**Purpose:**

To evaluate the predictive value of serum cystatin C and indirect bilirubin (IBIL), both individually and in combination, for sight-threatening diabetic retinopathy (STDR) in type 2 diabetes mellitus (T2DM).

**Methods:**

Retrospective cross-sectional study. Comprehensive demographic, clinical, and laboratory data were collected. Statistical analyses included between-group comparisons, univariable and multivariable logistic regression, and receiver operating characteristic (ROC) curve analysis. Patients were stratified into cystatin C tertiles to examine dose-response relationships.

**Results:**

151 T2DM patients (79 non-STDR, 72 STDR) recruited from two clinical centers. STDR patients showed significantly higher cystatin C (1.10 [0.91, 1.31] vs. 0.88 [0.80, 1.01] mg/L, P < 0.001) and lower IBIL levels (6.90 ± 3.89 vs. 9.72 ± 4.98 μmol/L, P < 0.001). Multivariable analysis confirmed cystatin C (OR = 1.342 per 0.1 mg/L increase, 95% CI 1.111-1.621, P = 0.002) and IBIL (OR = 1.139 per μmol/L decrease, 95% CI 1.031-1.259, P = 0.011) as independent STDR predictors. The highest cystatin C tertile had 7.576-fold increased STDR odds (95% CI 2.560-22.419, P < 0.001). ROC analysis showed cystatin C (>1.025 mg/L) predicted STDR with area under the receiver operating characteristic curve (AUROC)=0.737, while IBIL (<6.09 μmol/L) had AUROC = 0.667. Combination strategies provided flexible performance: “OR” rule (either high cystatin C or low IBIL) achieved 80.6% sensitivity, while “AND” rule (both high cystatin C and low IBIL) reached 94.9% specificity.

**Conclusions:**

Serum cystatin C and IBIL are independent predictors of STDR in T2DM. Their combination offers a flexible screening approach—achieving either high sensitivity or high specificity—providing a practical tool for risk stratification and early detection of STDR.

## Introduction

The growing burden of diabetes mellitus (DM) worldwide is a major public health concern, driven by its high prevalence and associated complications (1). Reports from the World Health Organization (WHO) and International Diabetes Federation indicate a rise in global diabetes prevalence from 537 million adults in 2021 to a projected 783 million by 2045 (2). A major complication, diabetic retinopathy (DR), currently accounts for 4.8% of blindness worldwide and is projected to cause blindness in over 10 million people by 2030 (2). The significant burden of DR—which affects approximately 30% of the diabetic population, with sight-threatening complications (STDR) developing in 10%—represents a substantial public health challenge (3, 4). Consequently, it is imperative to identify and validate new biomarkers for early-stage detection.

Despite its widespread use, fundoscopy for DR screening remains resource-intensive and limited by inter-observer variability. As DR pathogenesis involves systemic inflammation and metabolic dysregulation, blood-based biomarkers such as cystatin C hold significant potential for identifying STDR. Cystatin C, a muscle mass-independent marker of renal function, has been linked to microvascular complications (5). International multicenter studies have validated elevated cystatin C as a predictor of STDR risk, demonstrating high diagnostic accuracy (6, 7).

On the other hand, oxidative stress—driven by an imbalance between reactive oxygen species production and antioxidant capacity—plays a key role in DR pathogenesis, bilirubin serves as a potent endogenous antioxidant that protects retinal tissue by neutralizing free radicals, and large-scale longitudinal studies have consistently confirmed that lower bilirubin levels are significantly associated with increased DR risk and severity (8–11). Recent evidence from 6,993 hospitalized type 2 DM (T2DM) patients further identified indirect bilirubin (IBIL) as a particularly valuable predictor (12).

In other words, cystatin C, a marker of renal function and systemic microvascular dysfunction, reflects chronic inflammation and endothelial damage—processes central to DR pathogenesis. Conversely, IBIL is a potent endogenous antioxidant; low levels signify compromised antioxidant capacity and heightened oxidative stress, a key driver of retinal vascular injury in diabetes. Although prior evidence has linked each biomarker individually to STDR, the predictive utility of their combination has not been reported—an important consideration given that both markers are routinely measured, inexpensive, and widely available, unlike other emerging candidates that require specialized assays or lack validation. Therefore, we hypothesized that the combination of serum cystatin C and IBIL could provide a flexible screening strategy. This study aimed to evaluate their individual and combined predictive value for STDR in a T2DM study population.

## Materials and methods

### Participants and assessments

This two-center, retrospective, cross-sectional study enrolled patients with T2DM, with or without STDR, between January 2022 and April 2023. The study protocol received approval from the Institutional Review Boards of two clinical centers, and was conducted in accordance with the ethical principles of the Declaration of Helsinki. Written informed consent was waived due to the retrospective nature of the study, and all patient data were anonymized and de-identified prior to analysis.

Demographic information (age, sex) and clinical history (diabetes duration, anemia, hypertension) were extracted from the electronic medical record system. Laboratory parameters assessed at enrollment included glycated hemoglobin A1c (HbA1c), estimated glomerular filtration rate (eGFR), serum creatinine, lipid profile, hemoglobin, cystatin C, total bilirubin, IBIL, direct bilirubin, and urinary microalbumin-to-creatinine ratio (ACR).

The inclusion criteria for eligible T2DM patients in this study were as follows: (1) initial clinical visit between January 2022 and April 2023, and (2) availability of at least one spectral-domain optical coherence tomography (SD-OCT) scan (Heidelberg Engineering, Heidelberg, Germany or Topcon, Tokyo, Japan) along with one color fundus photograph (acquired using TRC-50DX or Canon CR-2 systems). Exclusion criteria included: (1) age ≤18 years; (2) diagnosis of type 1 DM or other forms of DM; (3) chronic kidney disease stage 3 or higher, or an eGFR <60 ml/(min·1.73 m²); (4) hypertensive nephropathy confirmed by renal biopsy; and (5) significant media opacity preventing adequate fundus evaluation. For patients with asymmetric disease involvement in both eyes, the more severely affected eye was included in the analysis. All participants self-identified as Han Chinese.

### Grouping, diagnosis and definitions

Patients were categorized into two groups: Non-STDR and STDR. Non-STDR group included individuals with no DR, mild non-proliferative DR (NPDR), or moderate NPDR in the absence of diabetic macular edema (DME). STDR group comprised patients with severe NPDR (defined by the 4:2:1 rule: more than 20 hemorrhages in each of four quadrants, venous beading in two quadrants, or intraretinal microvascular abnormalities in one quadrant), proliferative DR (PDR; defined by neovascularization, vitreous/preretinal hemorrhage, or tractional retinal detachment), and/or the presence of DME (13). The diagnosis of DR, mild-moderate NPDR, severe NPDR, and PDR was based on standardized color fundus photography, interpreted according to the International Clinical Diabetic Retinopathy Disease Severity Scale (13). DME was diagnosed using SD-OCT, defined as a retinal thickening (≥250 µm) within one disc diameter of the center of the macula or definite hard exudates in this region, consistent with Early Treatment Diabetic Retinopathy Study guidelines (14).

The diagnosis of T2DM required either a physician-documented history of T2DM or current treatment with oral hypoglycemic agents (with or without insulin therapy). Hypertension was defined as a documented history of arterial hypertension or current use of at least one antihypertensive medication. Despite known gender variation in hemoglobin levels, anemia (defined by gender-specific cutoffs: <120 g/L for males and <110 g/L for females) was evaluated as a candidate predictor.

### Standardized protocols, laboratory methods, and ophthalmic imaging

Both centers (Beijing Tsinghua Changgung Hospital and China-Japan Friendship Hospital) followed identical, pre-defined protocols for data abstraction from electronic medical records, ensuring consistency in variable definition and collection. All laboratory assays (including serum cystatin C, bilirubin fractions, and lipid profiles) were performed at each center’s clinical laboratory using standardized, commercially available assays that are regularly calibrated and subject to external quality assessment. While equipment platforms may differ between centers, all assays met national clinical laboratory standards. For fundus photography and SD-OCT, both centers used certified devices. Image grading was performed by two experienced retina specialists (SC. Si and YJ Yan) at each center, and any discrepancies were resolved by consensus.

### Statistical analysis

All statistical analyses were performed using SPSS Statistics for Windows (Version 25.0; IBM Corp.). Continuous variables are summarized as mean ± standard deviation or median with interquartile range, based on their distribution as assessed by the Shapiro-Wilk test. Categorical variables are presented as frequencies and percentages.

Between-group differences in demographic, clinical, and laboratory parameters were analyzed using Student’s t-test, the Mann-Whitney U test, or the χ² test, as appropriate. Subsequently, variables with significant between-group differences (*P* < 0.05) were subject to univariable logistic regression analysis to identify STDR predictors. To mitigate multicollinearity, a Pearson’s correlation coefficient of |r|>0.5 was set as an *a priori* criterion for high collinearity; in such cases, only the variable with the lower *P* value from the between-group comparison was retained for modeling. Significant predictors from the univariable models (*P* < 0.05) were then included in a multivariable model, with results expressed as odds ratios (ORs) and 95% confidence intervals (CIs).

Thereafter, patients were stratified into tertiles based on serum cystatin C levels to ensure adequate sample distribution and to examine potential non-linear risk trends. Those in the upper two tertiles (T2 and T3) were compared to the lowest tertile (T1). Finally, receiver operating characteristic (ROC) curve analysis was conducted to evaluate the predictive performance of the identified predictors (cystatin C and IBIL) for STDR. The areas under the ROC curves (AUROCs) were compared, and the combined predictive value of serum cystatin C and IBIL was assessed. A two-sided *P* value<0.05 was considered statistically significant.

## Results

### Demographic, clinical, and laboratory characteristics

A total of 151 patients with T2DM (mean duration 12.53 ± 8.00 years) were included in this study, comprising 82 males and 69 females. Based on their DR severity, 79 patients were classified as non-STDR and 72 as STDR. The study population ranged in age from 22 to 86 years, with a mean age of 57.23 ± 12.57 years. Among the 72 STDR patients, 25 (34.72%) presented with PDR and 26 (36.11%) with DME.

Comparative analysis demonstrated significant differences between the non-STDR and STDR groups in eight key parameters: diabetes duration (10.95 ± 7.62 vs. 14.56 ± 8.09 years, *P* = 0.010), anemia (15.19% vs. 37.50%, *P* = 0.002), hemoglobin levels (138.50 ± 17.37 vs. 130.51 ± 19.51 g/L, *P* = 0.009), creatinine (64 [52, 77] vs. 69 [54, 91] μmol/L, *P* = 0.026), cystatin C (0.88 [0.80, 1.01] vs. 1.10 [0.91, 1.31] mg/L, *P* < 0.001), total bilirubin (13.08 ± 5.81 vs. 9.74 ± 4.68 μmol/L, *P* < 0.001), IBIL (9.72 ± 4.98 vs. 6.90 ± 3.89 μmol/L, *P* < 0.001), and urinary ACR (3.91 [1.19, 6.67] vs. 5.00 [1.57, 22.72] mg/mmol, *P* = 0.039). In contrast, the groups were comparable in terms of sex distribution, age, and other measured variables (all *P*>0.05). The complete demographic, clinical, and laboratory characteristics of all recruited T2DM patients are summarized in **Table 1**.

**Table 1:** Demographic, clinical, and laboratory characteristics of all recruited T2DM patients.

Baseline characteristics	Grouping		All	*P* value
	Non-STDR	STDR		
Clinical findings	N=79	N=72	N=151	
Male	42 (53.16)	40 (55.56)	82 (54.30)	0.768
Age, y	58.35±12.71	56.00±12.38	57.23±12.57	0.252
PDR	0 (0)	25 (34.72)	–	–
DME	0 (0)	26 (36.11)	–	–
Diabetes of≥10 years	38 (48.10)	43 (59.72)	81 (53.64)	0.153
Diabetes duration, y	10.95±7.62	14.56±8.09	12.53±8.00	0.010*
Hypertension	43 (54.43)	46 (63.89)	89 (58.94)	0.238
Hypertension of≥10 years	21 (26.58)	19 (26.39)	40 (26.49)	0.979
Hypertension duration,^#^ y	10.55±10.73	13.50±12.78	11.91±11.74	0.271
CKD 2	48 (60.76)	41 (56.94)	89 (58.94)	0.634
Anemia	12 (15.19)	27 (37.50)	39 (25.83)	0.002*
Blood tests				
Hemoglobin, g/L	138.50±17.37	130.51±19.51	134.67±18.80	0.009*
HbA1c, %	8.38±2.09	8.27±2.09	8.33±1.97	0.741
TC, mmol/L	4.67±1.08	4.48±1.17	4.58±1.13	0.292
Triglyceride, mmol/L	1.72±1.00	1.77±1.59	1.74±1.30	0.823
LDL.C, mmol/L	2.90±0.91	2.67±0.93	2.79±0.92	0.145
HDL.C, mmol/L	1.21±0.28	1.21±0.31	1.21±0.29	0.874
eGFR, mL/min/1.73m^2^	95.93 (86.70, 106.40)	93.95 (73.48, 102.00)	95.59 (82.21, 105.83)	0.112
Creatinine, μmol/L	64 (52, 77)	69 (54, 91)	66.9 (53.7, 80.3)	0.026*
Cystatin C, mg/L	0.88 (0.80, 1.01)	1.10 (0.91, 1.31)	0.96 (0.83, 1.13)	<0.001**
Total bilirubin, μmol/L	13.08±5.81	9.74±4.68	11.49±5.55	<0.001**
IBIL, μmol/L	9.72±4.98	6.90±3.89	8.38±4.70	<0.001**
Direct bilirubin, μmol/L	3.36±2.46	2.84±1.74	3.11±2.16	0.139
Urinary ACR, mg/mmol	3.91 (1.19, 6.67)	5.00 (1.57, 22.72)	4.07 (1.20, 7.58)	0.039*

ACR, urinary microalbumin to creatinine ratio; CKD, chronic kidney disease; DME, diabetic macular edema; eGFR, estimated glomerular filtration rate; HbA1c, glycated hemoglobin; HDL.C, high density lipoprotein cholesterol; IBIL, indirect bilirubin; LDL.C, low density lipoprotein cholesterol; PDR, proliferative diabetic retinopathy; STDR, sight-threatening diabetic retinopathy; T2DM, type 2 diabetes mellitus; TC, total cholesterol. *= *P*<0.05; **= *P*<0.001.

Data were presented as mean±standard deviation, median (interquartile range) or no. (%). Between-group differences were analyzed using Student's t-test, the Mann-Whitney U test, or the χ² test, as appropriate. *P*<0.05 was considered to be statistically significant. Bold values denote statistical significance.

# "Hypertension duration" refers specifically to the length of time since diagnosis among patients with hypertension (N=89).

### Logistic regression analysis for predicting STDR in T2DM

Pearson’s bivariate correlation analysis identified significant collinearity (|r| > 0.5) between several variable pairs: cystatin C and creatinine (r=0.918, *P* < 0.001), cystatin C and urinary ACR (r=0.534, *P* < 0.001), hemoglobin levels and anemia (r=-0.652, *P* < 0.001), and IBIL with total bilirubin (r=0.925, *P* < 0.001). To mitigate potential multicollinearity, only the variable with the lower *P*-value from the between-group comparison was retained for subsequent univariable logistic regression analysis. Accordingly, creatinine, urinary ACR, hemoglobin, and total bilirubin were excluded from further modeling.

**Figure 1:**
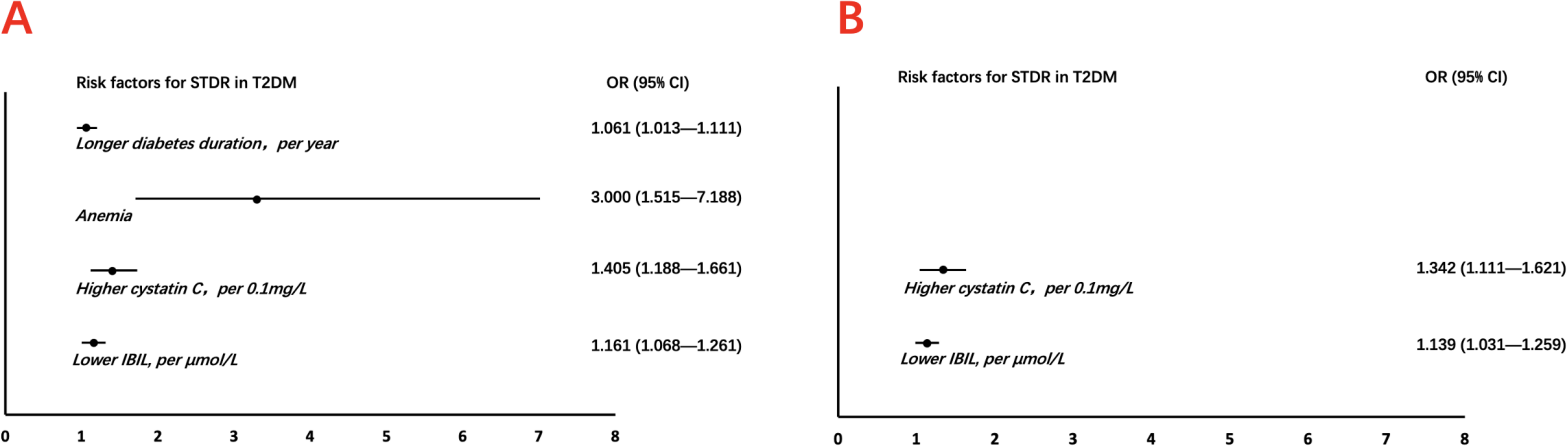
Risk factors predicting STDR in T2DM. **(A)** Univariable logistic regression analysis identified longer diabetes duration, anemia, elevated cystatin C levels, and reduced IBIL levels as significant predictors of STDR. **(B)** Multivariable analysis confirmed that elevated cystatin C and reduced IBIL remained independent risk factors for STDR after adjustment. CI, confidence interval; IBIL, indirect bilirubin; OR, odds ratio; STDR, sight-threatening diabetic retinopathy; T2DM, type 2 diabetes mellitus.

In the univariable analysis, longer diabetes duration (OR = 1.061 per year, 95% CI 1.013–1.111, *P* = 0.012), presence of anemia (OR = 3.300, 95% CI 1.515–7.188, *P* = 0.003), higher cystatin C levels (OR = 1.405 per 0.1 mg/L, 95% CI 1.188–1.661, *P* < 0.001), and lower IBIL levels (OR = 1.161 per μmol/L decrease, 95% CI 1.068–1.261, *P* < 0.001) were significantly associated with STDR. However, in the multivariable-adjusted model, only higher cystatin C (OR = 1.342 per 0.1 mg/L, 95% CI 1.111–1.621, *P* = 0.002) and lower IBIL levels (OR = 1.139 per μmol/L decrease, 95% CI 1.031–1.259, *P* = 0.011) remained independently associated with STDR (**Table 2** and **Figure 1**).

**Table 2:** The univariable and multivariable logistic regression analysis for STDR in T2DM.

Potential predictors	Univariable logistic analysis logistic analysis			Multiple logistic analysis regression		
	OR	95% CI	*P* value	OR	95% CI	*P* value
Diabetes duration, per year increase	1.061	1.013-1.111	0.012*	1.052	0.999-1.109	0.056
Anemia, yes vs. no	3.300	1.515-7.188	0.003*	1.097	0.384-3.134	0.863
Cystatin C, per 0.1mg/L increase	1.405	1.188-1.661	<0.001**	1.342	1.111-1.621	0.002*
IBIL, per μmol/L decrease	1.161	1.068-1.261	<0.001**	1.139	1.031-1.259	0.011*

CI, confidence interval; IBIL, indirect bilirubin; OR, odds ratio; STDR, sight-threatening diabetic retinopathy; T2DM, type 2 diabetes mellitus. * = *P*<0.05; **= *P*<0.001.

*P*<0.05 was considered to be statistically significant. Bold values denote statistical significance.

### STDR risk trend across cystatin C tertiles in T2DM

**Figure 2:**
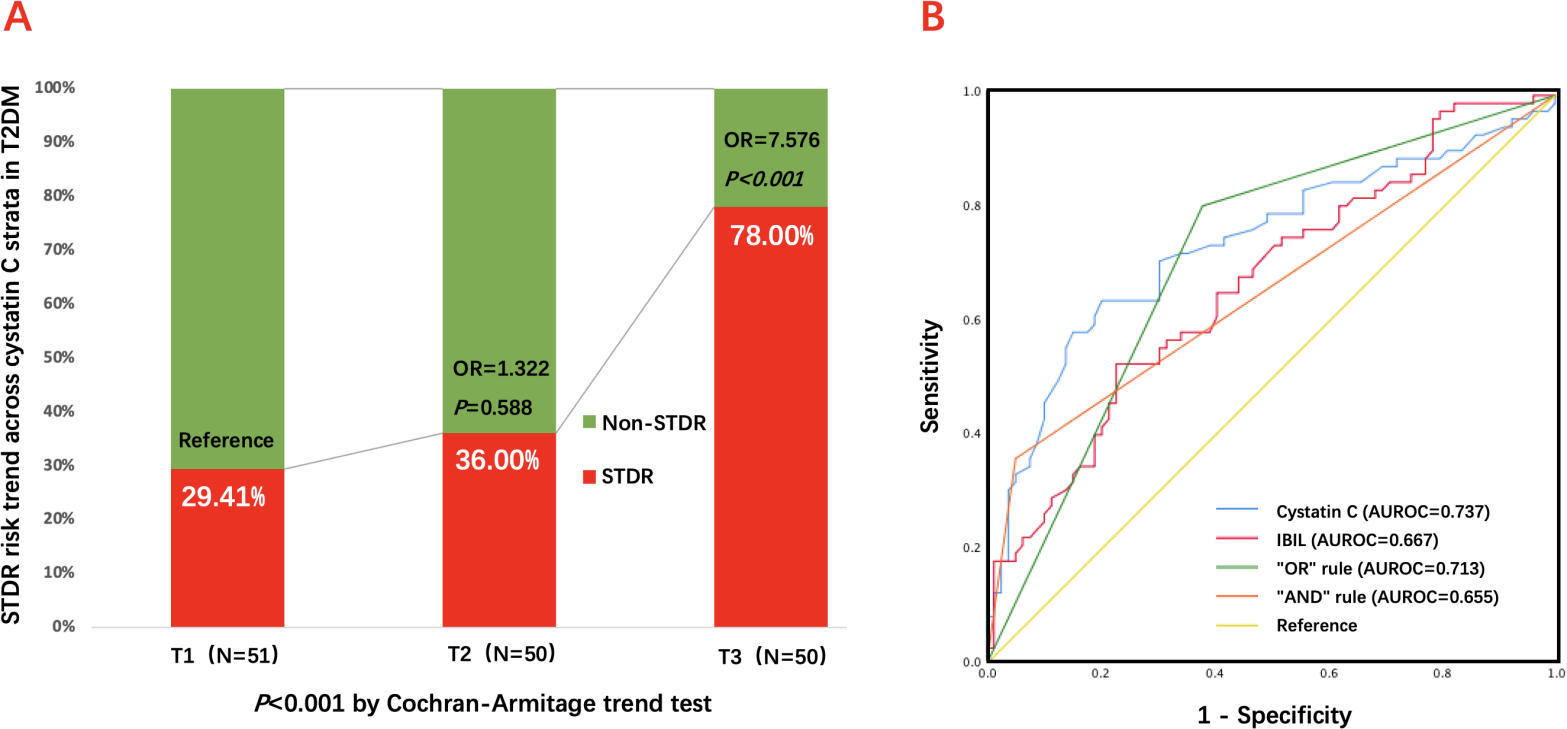
Predictive performance of cystatin C and IBIL, alone and in combination, for STDR in T2DM. **(A)** STDR risk increased significantly across ascending cystatin C tertiles (*P* < 0.001, Cochran–Armitage trend test). **(B)** ROC curves illustrate the discrimination performance of cystatin C, IBIL, and their combination for STDR. Cutoffs were derived from ROC analysis: high cystatin C was defined as >1.025 mg/L and low IBIL as <6.09 μmol/L. Combination rules were applied as follows: “OR” rule (high cystatin C or low IBIL) and “AND” rule (high cystatin C and low IBIL). AUROC, area under the receiver operating characteristic curve; IBIL, indirect bilirubin; OR, odds ratio; ROC, receiver operating characteristic; STDR, sight-threatening diabetic retinopathy; T2DM, type 2 diabetes mellitus.

To assess potential non-linear, dose-response relationships between cystatin C and STDR risk, patients were categorized into tertiles based on serum cystatin C levels (T1: n=51; T2: n=50; T3: n=50). A significant increasing trend in STDR incidence was observed across ascending tertiles (T1: 29.41%; T2: 36.00%; T3: 78.00%; *P* < 0.001 by Cochran-Armitage trend test) (**Table 3** and **Figure 2**).

**Table 3:** STDR incidence across cystatin C strata in T2DM.

Strata	Grouping		*P* value
	Non-STDR (N = 79)	STDR (N = 72)	
Serum cystatin C, mg/L			**<0.001**
Tercentile range (T1: n=51), No. (%)	36 (70.59)	15 (29.41)	
Tercentile range (T2: n=50), No. (%)	32 (64.00)	18 (36.00)	
Tercentile range (T3: n=50), No. (%)	11 (22.00)	39 (78.00)	

STDR, sight-threatening diabetic retinopathy; T2DM, type 2 diabetes mellitus.

*P*<0.05 was considered to be statistically significant. Bold values denote statistical significance.

Using T1 as the reference, univariable logistic regression showed that the T3 group had a significantly higher STDR risk (OR = 8.509, 95% CI 3.459–20.935, *P* < 0.001), while the T2 group showed no significant difference (OR = 1.350, 95% CI 0.586–3.110, *P* = 0.481). In the multivariable model adjusted for diabetes duration, anemia, and IBIL, both the T3 cystatin C level (OR = 7.576, 95% CI 2.560–22.419, *P* < 0.001) and lower IBIL (OR = 1.125 per μmol/L decrease, 95% CI 1.012–1.252, *P* = 0.008) remained independent risk factors for STDR (**Table 4**).

**Table 4:** Trend in STDR risk across cystatin C strata in T2DM.

Potential predictors	Univariable logistic analysis			Multiple logistic analysis regression		
	OR	95% CI	*P* value	OR	95% CI	*P* value
Diabetes duration, per year increase	–	–	–	1.039	0.984-1.098	0.163
Anemia, yes vs. no	–	–	–	1.314	0.474-3.639	0.600
IBIL, per μmol/L decrease	–	–	–	1.125	1.012-1.252	0.008*
Serum cystatin C, mg/L						
Tercentile range (T1: n=51)	Reference			Reference		
Tercentile range (T2: n=50)	1.350	0.586-3.110	0.481	1.322	0.481-3.629	0.588
Tercentile range (T3: n=50)	8.509	3.459-20.935	<0.001**	7.576	2.560-22.419	<0.001**

CI, confidence interval; IBIL, indirect bilirubin; OR, odds ratio; STDR, sight-threatening diabetic retinopathy; T2DM, type 2 diabetes mellitus. * = *P*<0.05; **= *P*<0.001.

*P*<0.05 was considered to be statistically significant. Bold values denote statistical significance.

### Incremental predictive value of combining cystatin C with IBIL for STDR

The ROC analysis demonstrated that a high cystatin C level predicted STDR with an AUROC of 0.737 (95% CI 0.655–0.819, *P* < 0.001), exhibiting 60.5% sensitivity and 79.2% specificity at an optimal cut-off value of 1.025 mg/L. A low IBIL level predicted STDR with an AUROC of 0.667 (95% CI 0.581–0.753, *P* < 0.001), showing 46.5% sensitivity and 85.4% specificity at a cut-off value of 6.09 μmol/L. When combining the markers using an “OR” rule (Cystatin C >1.025 mg/L or IBIL <6.09 μmol/L), sensitivity substantially increased to 80.6% for detecting STDR (AUROC = 0.713, 95% CI 0.630–0.796, *P* < 0.001), albeit with reduced specificity (62.0%). Conversely, using an “AND” rule (Cystatin C >1.025 mg/L and IBIL <6.09 μmol/L) markedly increased specificity to 94.9% (AUROC = 0.655, 95% CI 0.567–0.744, *P* = 0.001), though sensitivity was low (36.1%). Complete ROC results are provided in **Table 5** and **Figure 2**.

**Table 5:** Predictive performance of cystatin C and IBIL, alone and in combination.

Risk factors	ROC curve for STDR logistic analysis regression					
	Cut off	AUROC	Sensitivity	Specificity	95% CI	*P* value
Higher cystatin C, mg/L	1.025	**0.737**	0.605	0.792	**0.655-0.819**	<0.001**
Lower IBIL, μmol/L	6.09	0.667	0.465	0.854	0.581-0.753	<0.001**
Cystatin C>1.025 mg/L ***or*** IBIL<6.09 μmol/L		0.713	**0.806**	0.620	0.630-0.796	<0.001**
Cystatin C>1.025 mg/L ***and*** IBIL<6.09 μmol/L		0.655	0.361	**0.949**	0.567-0.744	0.001*

AUROC, area under the receiver operating characteristic curve; CI, confidence interval; IBIL, indirect bilirubin; ROC, receiver operating characteristic; STDR, sight-threatening diabetic retinopathy; T2DM, type 2 diabetes mellitus. **= *P*<0.001.

*P*<0.05 was considered to be statistically significant. Bold values denote statistical significance.

## Discussion

In the present study, higher serum cystatin C (OR = 1.342 per 0.1 mg/L increase, 95% CI 1.111–1.621, P = 0.002) and lower IBIL (OR = 1.139 per μmol/L decrease, 95% CI 1.031–1.259, P = 0.011) were identified as independent and significant predictors of STDR in T2DM patients. The observed OR for serum cystatin C aligns with values reported in previous literature (e.g., OR = 1.13 in the UK and 1.38 in India) (7). However, the association for decreased IBIL was slightly weaker in our study (OR = 1.139 for STDR) compared to that reported by Xu Man-Rong et al. (OR = 1.208 for any DR) (9). This observed discrepancy likely arises from multiple factors. Although the difference in outcome definition—predicting STDR rather than any DR—represents one key consideration, other methodological and population-level distinctions may also contribute. The more stringent STDR outcome used in our study may attenuate the predictive strength of an individual biomarker such as IBIL, compared to its ability to discriminate any stage of DR. Additionally, differences in study population characteristics—including ethnicity background, lifestyle, comorbidity profiles, and the underlying distribution of DR severity—may further explain the variation in effect sizes (6, 7). Despite these contextual differences, both serum cystatin C and IBIL remain readily measurable and cost-effective biomarkers with clear potential to supplement or enhance existing DR screening paradigms, which remain largely reliant on resource-intensive imaging and specialist evaluation.

On univariable analysis, both longer diabetes duration (OR = 1.061 per year, 95% CI 1.013–1.111, *P* = 0.012) and anemia (OR = 3.300, 95% CI 1.515–7.188, *P* = 0.003) showed significant associations with STDR. The result for anemia corroborates earlier work by Yin W et al. indicating an inverse relationship between hemoglobin concentration and DR risk (15). In the present study, however, we excluded patients with an eGFR <60 ml/min/1.73 m², which may have contributed to the lack of an independent association between anemia and STDR after multivariable adjustment. A recent retrospective study of 2,570 hospitalized patients with T2DM demonstrated that anemia is a risk factor for rapid eGFR decline, suggesting a potentially profound influence of anemia on renal function (16), and revealed that anemia may be associated to renal function more profoundly. Furthermore, Jin et al. (17) proposed that the relationship between anemia and DR may be mediated by concomitant changes in serum bilirubin levels. This offers a plausible explanation for our finding that anemia was not independently associated with STDR in a model that included both IBIL and anemia.

As demonstrated by the tertile analysis in this study, a graded association was observed between increasing cystatin C tertiles and elevated risk of STDR (T3 vs. T1: OR = 8.509, 95% CI 3.459–20.935, *P* < 0.001). These findings reinforce cystatin C as a robust predictor of microvascular damage. The tertile-based stratification provides a clinically interpretable risk gradient, moving beyond a simple binary association toward a more nuanced risk assessment. Thus, cystatin C should be regarded not merely as a renal function marker (18), but also as a potential indicator of systemic microvascular dysfunction (19–21). Its elevation may reflect shared pathophysiological pathways underlying both renal and retinal damage, such as chronic inflammation and endothelial dysfunction, which are central to the progression of DR (5). These results are consistent with previous literature, including studies by Pramodkumar Thyparambil et al. (2023), Gurudas Sarega et al. (2022), and He R et al. (2013), which have also validated the role of cystatin C in predicting STDR (6, 7, 19).

The observed inverse relationship between IBIL and STDR underscores the role of IBIL as a potent endogenous antioxidant. Conversely, lower IBIL levels may reflect a compromised antioxidant defense system, resulting in heightened oxidative stress in the retina—a key mechanism driving vascular injury, inflammation, and breakdown of the blood-retinal barrier in DR (22, 23). This finding aligns with the broader consensus established in longitudinal studies, such as those by Ding et al. (2020) and Liu et al. (2018), which identify low bilirubin as a significant risk factor for DR (8, 11). Furthermore, our study highlights the novel insight that IBIL—rather than total bilirubin—serves as a key predictor of STDR, a conclusion consistent with the recent report by Xu Man-Rong et al. (9).

A key innovation of our study lies in the integrated application of cystatin C and IBIL, with distinct clinical implications derived from different combination rules. The application of “OR” and “AND” decision rules provide a flexible and practical screening strategy. Specifically, the “OR” rule (cystatin C >1.025 mg/L or IBIL <6.09 μmol/L) provides high sensitivity (80.6%), serving as an effective first-line screening tool to rule out STDR in broad populations. This approach is particularly suitable for medically underserved areas, suburban primary care clinics, and non-ophthalmic outpatient settings (e.g., general practice and endocrinology), where it enables efficient identification of high-risk individuals for specialist referral. In contrast, the “AND” rule (cystatin C >1.025 mg/L and IBIL <6.09 μmol/L) achieves high specificity (94.9%), functioning as a powerful “rule-in” tool that assists clinicians in identifying patients with a high probability of STDR, thus aiding definitive diagnosis and facilitating urgent treatment planning. Together, this flexible, dual-marker strategy supports tailored screening aligned with varying clinical objectives and resource availability.

However, several limitations of this study should be considered. First, the cross-sectional design of this study precludes causal inference, and our findings should be interpreted as demonstrating associations rather than temporal or causal relationships. While our results are consistent with prior longitudinal studies (e.g., Ding et al., 2020; Liu et al., 2018) (8, 11) that support the biological plausibility of these associations, prospective cohort studies with longitudinal follow-up and repeated biomarker measurements are essential to establish whether elevated cystatin C and decreased IBIL precede the development or progression of STDR. Second, the relatively modest sample size (n=151) comprising only Han Chinese individuals may limit the generalizability of our findings. Validation through larger, multi-ethnic prospective cohorts is needed to confirm these results and establish universally applicable cut-off values. Third, we could not adjust for several potential confounders due to data unavailability. Although hypertension diagnosis was included as a covariate, detailed longitudinal blood pressure measurements and antihypertensive medication adherence were not recorded, which may influence both biomarker levels and DR progression. Similarly, we lacked granular data on specific medications (e.g., statins or RAS inhibitors) (24, 25) that may have retinoprotective or anti-inflammatory effects. Additionally, unmeasured lifestyle factors—including dietary habits, physical activity, weight changes, and smoking status—could affect bilirubin metabolism and DR risk. Future studies should incorporate comprehensive medication histories and lifestyle assessments to better account for these residual confounders. Fourth, while our composite STDR outcome aligns with the clinical objective of identifying patients requiring urgent ophthalmic intervention, the current sample size was underpowered for robust subgroup analyses. Consequently, we were unable to definitively assess whether the predictive performance of cystatin C and IBIL differs across specific DR phenotypes (e.g., between NPDR and PDR, or between DME and non-DME). Future large-scale, prospective studies are warranted to explore potential variations in biomarker utility according to distinct stages and pathological features of DR. Fifth, although this study was conducted at two clinical centers, the retrospective design means that residual center effects cannot be entirely excluded. However, we implemented standardized data collection protocols and rigorous quality assurance measures across both sites to minimize potential systematic bias. Future prospective studies should consider incorporating centralized laboratory analyses and standardized reading centers for ophthalmic imaging to further reduce inter-site variability and enhance the robustness of the findings.

In conclusion, the combination of cystatin C and IBIL represents a promising, accessible, and cost-effective strategy for enhancing risk stratification in T2DM patients, potentially enabling earlier clinical intervention and improved vision preservation worldwide. Importantly, the “OR” and “AND” combination rules employed here offer flexible, clinically actionable strategies that leverage the complementary strengths of each biomarker, enabling either high-sensitivity screening or high-specificity confirmation depending on the clinical context. However, this study did not assess other potential biomarkers (e.g., specific inflammatory markers), a pragmatic decision guided by data availability from electronic medical records. Future research should investigate whether incorporating such biomarkers into the cystatin C-IBIL panel offers incremental predictive value. Additionally, efforts should prioritize the integration of these serum biomarkers with retinal imaging data and artificial intelligence algorithms to develop more comprehensive and automated risk prediction models.

## Data Availability

The raw data supporting the conclusions of this article will be made available by the authors, without undue reservation.
